# Mechano-Growth Factor: an important cog or a loose screw in the repair machinery?

**DOI:** 10.3389/fendo.2012.00131

**Published:** 2012-11-01

**Authors:** Barbara Zabłocka, Paul H. Goldspink, Geoffrey Goldspink, Dariusz C. Górecki

**Affiliations:** ^1^Molecular Biology Unit, Mossakowski Medical Research Centre PASWarsaw, Poland; ^2^Department of Physiology and Cardiovascular Center, Medical College of WisconsinWI, USA; ^3^Professor Emeritus, Department of Surgery and Interventional Medicine, University College LondonLondon, UK; ^4^Institute of Biomedical and Biomolecular Sciences, University of PortsmouthPortsmouth, UK

Insulin-like growth factor I (IGF-1) is an important peptide synthesized in response to growth hormone stimulation. Alternative promoters and an elaborate alternative splicing regulated in a tissue- and developmentally-specific manner result in the production of several distinct isoforms of IGF-1 [reviewed in Gorecki et al. (2007); Matheny et al. (2010)]. The predominant form, IGF-1Ea is a circulating factor produced in the liver while IGF-1Eb and IGF-1Ec (Mechano-Growth Factor, MGF) are expressed in specific tissues in response to different, often pathological stimuli and appear to have some specific functions in these different tissues [reviewed in Aberg et al. (2006); Gorecki et al. (2007); Matheny et al. (2010)].

Unfortunately, the initial naming of these specific isoforms caused some confusion (Matheny et al., 2010), mainly because 3′-end splicing in rodents and humans results in slightly different reading frames and also due to the overlapping naming of the synthetic peptide domains (see below). We have consistently adopted the designations suggested in Hameed et al. (2003). Thus, the main human transcript (IGF-1Ea) has exon 4 spliced directly to exon 6. Inclusion of exon 5 results in two transcript variants: IGF-1Ec (also designated MGF) contains part of exon 5 spliced to exon 6, which results in a frame-shift and this mRNA is translated into an isoform with alternative 25 amino acids at the C-terminus. The third transcript variant (IGF-1Eb) extends further downstream of exon 5 but only the first 17 amino acids of this isoform are identical with those in the IGF-1Ec peptide (Siegfried et al., 1992).

IGF-1 isoforms are produced as a preprohormone containing a signal peptide at the amino-terminus. Removal of the signal peptide leaves the prohormone (mature peptide + E-domain), which is further cleaved to yield the mature peptide (70 amino acids) and the E-domain. Cleavage of the E-domain region occurs at a pentabasic-processing motif LysXXLys^68^XXArg^71^XXArgXXArg between the D and E-domains (Duguay et al., 1995). Disruption of this cleavage site does not impact secretion *per se*, but appears to modulate the activities and actions of the mature IGF-1 (Pfeffer et al., 2009; Barton et al., 2010). Interestingly, the existence of the different E-domain regions gives rise to unique isoform-specific gene expression profiles compared to those associated with mature IGF-1 (Barton et al., 2010). These data suggest the E-domains regulate aspects of IGF-1 actions whether in the prohormone form or independently upon cleavage. Given the existence of this cleavage site in all the isoforms and disruption modifies behavior, it is reasonable to hypothesize that the E-domain may have independent actions *in vivo*. Indeed, research to date has been directed at defining those actions in relation to MGF and the use of peptide mimetics have been insightful (see below).

Specific actions of IGF-1 isoforms have been identified in different tissues, including development, growth, repair, and maintenance of muscles. Skeletal muscle is a post mitotic tissue and cell division does not take place within muscle fibers once they are formed. During post-natal, post-damage and exercise-induced muscle growth the proliferating cells (myoblasts) required for these processes originate from the pool of muscle stem (satellite) cells. IGF-1 stimulates differentiation and, to a lesser degree, proliferation in cultured primary satellite cells. There is evidence of an autocrine/paracrine stimulation via IGF-1 (Goldspink, 2004; Hameed et al., 2004; Mourkioti and Rosenthal, 2005). Skeletal muscle stretch/overload results in the increase of the IGF-1 mRNA, particularly its specific IGF-1Ec (MGF) variant. In this system the specific effects of the synthetic E-domain peptide mimetic have been shown: satellite cells are activated to replicate by the MGF-24aa-E peptide (YQPPSTNKNTKSQRRKGSTFEEHK) (Kandalla et al., 2011) and they are prevented from going further until they fuse with the muscle fibers and when they adopt a myogenic program (Qin et al., 2012). During ageing, muscle produces less MGF transcript but if it is provided to myoblasts cultures taken from biopsies of muscle from an elderly person, these muscle cells are still able to replicate (Kandalla et al., 2011).

In the heart, there is temporal regulation of both MGF and IGF-IEa mRNA expression in response to ischemia associated myocardial infarction. MGF expression is induced within one hour and remains elevated for up to 8 weeks whereas the IGF-Ea appears after 4 days (Stavropoulou et al., 2009). These expression profiles suggest that the isoforms play distinct and separable roles in local adaptation aimed at mitigating tissue damage to augment cardiac output. Moreover, intracoronary delivery of the MGF-24aa-E peptide elicits myocardial protection and improves hemodynamic function to a greater extent than mature IGF-1 following myocardial infarction in sheep. As the analog does not have the IGF-1 receptor (IGF-1R) binding site, the mechanisms of action may differ (see below). Limited mechanistic findings suggested that the cellular protection conferred by the MGF-24aa-E peptide, was due to an inhibition of apoptosis in the infarct border zone (Carpenter et al., 2008). However, given the growing data showing effects of the MGF-24aa-E peptide on various precursor cell populations, it is an intriguing notion to speculate that similar effects may exist on cardiac precursor cell populations to aid in repair and recovery of the heart. It is well known the heart has a limited capacity for regeneration, but recent studies have shown the existence of resident populations of multipotent precursor cells (Segers and Lee, 2008). These cells exist in extremely low numbers but if stimulated, could provide an endogenous source for the different lineages (cardiac, endothelia, and vascular) needed to initiate regeneration. To this end, the MGF-24aa-E peptide has been shown to increase proliferation and migration of mesenchymal bone marrow derived stem cells, which have been proposed as a source of autologous stem cells for transplantation into the heart (Collins et al., 2010). In addition, the MGF-24aaE peptide appears to stimulate pro-angiogenic activities in human vascular endothelial cells (Deng et al., 2012). Thus, delivery of the MGF-24aa-E peptide could bestow potentially useful therapeutic actions at the level of vascular regeneration and collateralization, to restore blood flow in the heart following myocardial infarction.

Similarly, specific IGF-1 isoforms are important factors for brain development and function, are expressed in response to different types of injury and can reduce neuronal death (Dluzniewska et al., 2005; Beresewicz et al., 2010). Again, while both IGF-1Ea and IGF-1Ec (MGF) transcripts have been shown expressed during brain development, they show highly specific temporal distributions. Moreover, neonatal hypoxia and hypoxia-ischemia insults produce increased and prolonged expression of the IGF-1Ec (MGF) isoform only (Beresewicz et al., 2010). Furthermore, MGF but not IGF-1Ea was found overexpressed in the regenerating regions following global adult brain ischemia. In this model, the MGF-24aa-E peptide has also shown specific effects independent from the IGF-1R stimulation (Dluzniewska et al., 2005).

Furthermore, IGF-1 plays a complex role at the interface between neurons and the injured or damaged muscle. In a model of amyotrophic lateral sclerosis (ALS), a disease with loss of motor neurons and progressive muscle weakness, muscle overexpression of IGF-1 slowed the disease progression (Dobrowolny et al., 2005). While treatment with either full-length IGF-I or MGF expressing constructs resulted in improvements in this mouse model, significantly more motor neurons survived in MGF-treated mice (Riddoch-Contreras et al., 2009). While clinical trials with IGF-1 gave conflicting results, recent data indicate that this treatment might be effective in patients with less severe phenotypes (Saenger et al., 2012).

Mature IGF-1 acts *via* the IGF-1R. Binding induces phosphorylation of the receptor and activates the downstream effector cascades involving the MAP kinase, cyclin D, cdk4, c-fos, c-jun, calcium–calmodulin-dependent kinase and, *via* PI3-K, Akt pathways downstream effector genes. In addition, IGF-1 activity is modulated by its binding proteins (IGFBPs) and autoregulatory feedback loops aimed at regulating receptor mediated signaling. The aforementioned differential effects of IGF-1 isoforms appear to stem from the fact that the actions of the E-domains can be separated from receptor activation and show preferences with respect to the signal transduction pathways. This further supports the notion that synthetic equivalents of the E domains may function as independent biologically active peptides. Unfortunately, still little is known about the mechanism(s) of action and wider biological effects of these short peptides but data are starting to indicate distinct pathways may be involved. In case of the MGF-24aa-E peptide, one recent study demonstrated that PKC activity is needed for this peptide activation (translocation to nucleus) of NF-E2-related factor-2 (Nrf2), which in turn increases heme oxygenase-1 expression, a critical event in mediating neuroprotection of neurons from oxidative stress-induced apoptosis (Quesada et al., 2011). In another models, the MGF-24aa-E peptide appears to stimulate the MAPK\ERK-signaling pathway (Stavropoulou et al., 2009; Deng et al., 2012).

In view of the difference in their modes of action it appears possible to separate the broad activity of the mature IGF-1 peptide from the protective and pro-regenerative effects of its E-domain derivatives. Moreover, small peptides show good tissue penetration, can be inexpensively manufactured and modified to increase their stability. They are therefore good candidates for development into a therapeutic modality and, not surprisingly, such compounds attract attention of pharmaceutical industry.

Intriguingly, the MGF-24aa-E peptide has attracted considerable attention, but not from the mainstream pharmaceutical industry or the medical establishment but instead from the poorly regulated or outright illegal branches of pharma: the MGF-24aa-E peptide has become an important component of the readily available anabolic and doping products and there may be a number of reasons for this peculiar situation.

Firstly, there is controversy surrounding the endogenous production of short E-domain peptides. While the full length IGF-1Ec isoform has been identified using E-domain-specific antibodies in various experimental settings, the existence of the endogenously functioning E peptides has not been confirmed despite the fact that the equivalent synthetic E-domain peptide is effective (Tan et al., 2002; Dluzniewska et al., 2005). It could be therefore argued that the effects of the MGF-24aa-E mimetic are not related to the prohormone form containing this E domain. However, considering that endogenous expression of this isoform has been identified in specific tissues in which MGF-24aa-E peptide is efficacious, it seems an unlikely coincidence. Nevertheless, it does not clarify whether the endogenous E domain exerts its function as a prohormone before it is further cleaved into the mature IGF-1 peptide and the E-domain. Closely linked with this issue is the very important and still unanswered question of the mechanism of action of the E-domain and its peptide mimetics. All we really know is that it does not require the canonical binding site of the IGF-1R. This is supported by recent studies using the MGF-24aa-E peptide showing that the putative site of action of the MGF E-domain region maybe at the level of the nucleus (Quesada et al., 2011; Schönenberger et al., 2011; Peng et al., 2012). These data would further support cleavage of the E-domain region *in vivo* and its targeting to the nucleus, where it might exert intracrine actions to modify the canonical IGF-1 signaling *via* it's receptor at the cell membrane. This effect is not uncommon in the peptide hormone and growth factor family (Re and Cook, 2011).

In addition, the difficulty with identifying these short peptides may stem from the fact that molecules of IGF-1 aggregate at pH close to its isoelectric point (Torrado and Carrascosa, 2003) and this covalent aggregation is one of its main degradation pathways. If such aggregation involves the short E-domain peptides it could mask their presence in tissues. Moreover, studies with various synthetic E-domains have shown that they benefit from chemical stabilisation (Collins et al., 2010) and thus their endogenous equivalents are predicted to have a very short half-life. In fact they may be active at very low concentrations functioning in an autocrine/paracrine/intracrine fashion which would be expected for growth factors in the physiologic environment, further complicating detection of their endogenous processing.

Clearly more research is needed. But MGF seems to be a highly under-researched subject, particularly if one considers the likely applications of MGF in diseases with a great socioeconomic impact in aging populations (e.g., cardiovascular, sarcopenia, and neurodegeneration). This probably reflects the complexities of biopharmaceuticals development. These products, by the very nature of their origins face specific problems in terms of manufacture, storage, quantifiable standardization and, finally, their approval. This is usually associated with higher costs when compared with small molecule drugs. Industry often finds these costs unacceptable when facing possible patent right challenges based on slightly modified biosimilars and rapid emergence of market competition following patent expiry.

In an astonishing contrast, the MGF has been welcomed and widely accepted by the body building industry and numerous fitness enthusiasts. Body builders use this peptide alone or in combination with other anabolic compounds. Perfunctory survey of various discussion *fora*^1^ makes a chilling read for anybody who knows how little is yet known about the MGF. Detailed advice on administration regimes contains a mixture of in depth scrutiny of scientific literature and a hear-say following self-administration. Although its function is not fully understood and its efficacy in man is not proven, MGF is seen as a powerful tool to gain performance (Bachl et al., 2009). It is not surprising that the World Anti-Doping Agency has banned the use of MGF in sports. Recent study reporting MGF detection in two black market products (Esposito et al., 2012) clearly confirmed that MGF preparations are commercially available for use as doping. Even more ominously, illegal production of MGF mimetics and attempts to bypass the WADA ban may result in generation of analogs of unknown properties and potentially serious side effects.

The existence of IGF's has been studied for over five decades, when they have evolved as being growth hormone inter-mediators, endocrine, and autocrine/paracrine factors. This complex system, which is preserved through the vertebrate phylogeny, influences a remarkable array of cellular and organismal functions. Consequently, it may not be too surprising that additional levels of regulation and isoform function may exist due to the presence of the different E-domain regions in the splice variants and as such, present new challenges to fully understanding how the IGFs' work.

## Footnotes

^1^References to these sites are not provided for ethical reasons.
